# Willingness to use HIV pre-exposure prophylaxis and associated factors among men who have sex with men in Liuzhou, China

**DOI:** 10.1186/s12981-021-00374-8

**Published:** 2021-07-30

**Authors:** Yuansheng Fu, Akililu Alemu Ashuro, Xianxiang Feng, Ting Wang, Sichen Zhang, Dongqing Ye, Yinguang Fan

**Affiliations:** 1 Department of Epidemiology and Biostatistics, School of Public Health, Anhui Medical University, Hefei, China; 2 Anhui Province Key Laboratory of Major Autoimmune Diseases, Anhui Medical University, Hefei, China; 3 Liuzhou Center for Disease Control and Prevention, Liuzhou, China

**Keywords:** Pre-exposure prophylaxis, Men who have sex with men, HIV prevention, Willingness, China

## Abstract

**Background:**

HIV pre-exposure prophylaxis (PrEP) is a key component of HIV combination prevention strategies and has the potential to decrease the incidence of HIV in men who have sex with men (MSM). This study aimed to evaluate levels of PrEP acceptability and explore factors associated with willingness to use PrEP among MSM in Liuzhou, China.

**Methods:**

Between November 2017 and May 2019, a cross-sectional survey was conducted among 829 MSM. The study participants were recruited through a nonprobability sampling method. The participants’ demographics, HIV/AIDS knowledge, sexual behaviors, drug use and HIV test history were collected. Multivariate logistic regression was performed to identify factors associated with willingness to use PrEP.

**Results:**

A total of 829 MSM completed the survey, and 30.28% (95% CI 27.3–33.4) were willing to use PrEP. In multivariate logistic regression, factors associated with a higher willingness to use PrEP included Zhuang or Han ethnic origin, recruitment through peer introduction or gay venues, pursuit of a higher education level, previous HIV testing and oral sex with a man. Conversely, having casual sex in the past 6 months was associated with a lower willingness to use PrEP.

**Conclusions:**

MSM in Liuzhou reported a relatively low level of willingness to use PrEP. The results indicate the need for effective education, targeted intervention, and implementation strategies to promote PrEP acceptance among MSM.

## Background

Human immunodeficiency virus (HIV) causes acquired immune deficiency syndrome (AIDS) and has become a global public health burden, resulting in significant mortality and morbidity [1]. Although the HIV prevalence in China is relatively low, the number of newly diagnosed HIV cases continues to rise, with the majority of individuals who are infected HIV being unaware of their status. A comprehensive national policy and strategy for HIV prevention is expanding and improving access to HIV testing and treatment services, aiming to control the HIV epidemic in China to a low level. Men who have sex with men (MSM) are considered to be a key population at high risk of HIV infection. The HIV prevalence among MSM in China has increased significantly from 1.4% in 2005 to 8.0% in 2015 [2]. Additionally, over a quarter of new HIV diagnoses in China can be attributed to MSM [3]. Although the government provides a variety of HIV prevention methods, such as condoms, HIV and sexually transmitted infection (STI) testing and antiretroviral therapy (ART), the HIV epidemic among MSM remains uncontrolled, especially in the western part of the country [4, 5]. In view of these trends, there is an urgent need for China to implement new and effective HIV prevention measures among MSM.

HIV pre-exposure prophylaxis (PrEP), a new biomedical approach for HIV prevention using antiretroviral drugs (tenofovir disoproxil fumarate/emtricitabine, TDF/FTC) before HIV exposure among HIV-negative individuals, is increasingly recognized as the most promising HIV prevention strategy [6]. Indeed, clinical trials and cohort studies have demonstrated the safety and efficacy of oral PrEP in reducing the risk of HIV acquisition among MSM [7], heterosexual men and women [8] and people who inject drugs [9]. In 2015, the World Health Organization (WHO) recommended the use of oral PrEP as an additional prevention choice to all people at substantial risk of HIV infection, including MSM [10]. To date, the United States, France, Brazil, South Africa and several other countries have approved the use of PrEP among MSM. In China, the government carried out pilot work on HIV PrEP in seven provinces in 2019 and planned to develop national implementation guidelines [11, 12].

Although PrEP is obviously effective, its success ultimately depends on adoption by those who need it. A growing body of evidence from different time periods and locations has shown that MSM play an increasingly important role in China’s HIV/AIDS epidemic [13]. Recent national reports have shown that HIV transmission among MSM accounts for 21.4% to 23.4% of the newly identified HIV/AIDS cases in some areas in China [14], and research on the willingness to use PrEP among MSM has recently years been conducted in many countries [15–17]. Nevertheless, willingness varies across countries, which may limit the general application of existing studies. In addition, due to the sociodemographic, cultural, economic and structural factors that are likely to affect the acceptability of PrEP among MSM [3, 18, 19], it is important to understand how to implement PrEP according to local settings.

Liuzhou is a city with the highest prevalence of HIV infection in the Guangxi Zhuang Autonomous Region, which has the second-largest number of people living with HIV in South-Central China [20]. In Liuzhou, MSM mainly make contact through network platforms (e.g., websites, social media applications, etc.) and meet in bars, parks and bathhouses. Although nearly 1500 MSM each year have access to free HIV testing and prevention services at the Center for Disease Control and Prevention (CDC), medical institutions and local community-based organizations (CBOs), MSM rank second among newly identified HIV/AIDS cases in Liuzhou city [21]. However, there are no available data on willingness to use PrEP and factors among MSM in this region. Therefore, the aim of this study was to assess the level of willingness to use PrEP and to explore its relationship with sociodemographic factors, HIV/AIDS knowledge, sexual behaviors, drug use and HIV test history among MSM in Liuzhou. Findings on willingness to use PrEP and associated factors will provide a basis and guidance for the application and promotion of PrEP implementation among MSM in Liuzhou, China.

## Methods

### Participants and procedure

A cross-sectional study was conducted among MSM (cis-gender males who self-identified as gay/bisexual or reported having anal sex with another male) who lived in Liuzhou, China, from November 2017 to May 2019. Participants were recruited through a nonprobability sampling method, and the main methods of recruitment included the following: (1) identifying “seed” subjects through peer introduction and using the snowball sampling method to recruit other research subjects; (2) via network platform (i.e., websites and social media applications); and (3) recruited by trained and experienced peer educators from locations frequented by MSM, such as bars, parks and bathhouses. The inclusion criteria were as follows: (1) aged 15 years or older; (2) born biologically male; (3) had anal and/or oral intercourse with at least one man in the last 6 months; (4) HIV negative; and (5) residing in Liuzhou. HIV-positive individuals were excluded and referred to the local CDC. For eligible participants, an appointment will be made by phone, and they were briefly informed of the purpose of the study. Informed consent was obtained from each subject prior to enrolling them in the study. Each participant completed a face-to-face interview questionnaire in a private room at CBOs, which took approximately 20 min to complete. Respondents were compensated 50 RMB (approximately 7.5 US dollars) for their transportation cost and time spent on the survey. ID cards and addresses were cross-checked to avoid repetition. The design and implementation of the study protocol were completed by the faculty and graduate students of Anhui Medical University and approved by the Anhui Medical University ethics committee.

### Laboratory tests

Three milliliters of blood was collected and transported to the laboratory of the local CDC within 12 h. Plasma samples were screened for anti-HIV antibodies by enzyme-linked immunosorbent assay (ELISA; Beijing Modern Gaoda Biotechnology Co., Beijing, China), and positive tests were confirmed by an HIV-1/2 Western blot assay (HIV Blot 2.2 WBH; Genelabs Diagnostics, Singapore).

### Questionnaire

The validity of the questionnaire used in our study was ensured by expert argumentation and pilot tests. Before the formal survey, all questionnaire items were pretested with five MSM that met the study’s inclusion criteria. Revisions were made based on the pilot results and comments from experts. Subjects in the pilot test did not participate in the actual survey.

Demographic information collected included age, household registration, ethnicity, occupation, education level, marital status, personal monthly income, sexual orientation, history of sexually transmitted diseases (STDs) other than HIV, and recruitment source.

In all, HIV/AIDS knowledge was assessed by six questions, with two questions on mosquito bites and number of sex partners in HIV transmission, another two on condom use and lubricant use in HIV prevention, one on blood testing for HIV and one on HIV/AIDS treatment. There was one point assigned for a correct answer; 0 was assigned for a wrong answer or an answer of unknown or unsure. The total score for HIV literacy ranged from 0 to 6, and a score equal to or greater than 4 was classified as “high level of HIV/AIDS knowledge”; scores below 4 were classified as “low level of HIV/AIDS knowledge”.

Items for assessing sexual behaviors comprised two scales. The scale of homosexual behaviors was measured by the following questions: total number of male sexual partners, age at first anal sex with men, number of anal sex partners in the past month, condom use during anal sexual intercourse, oral sexual behavior with men, and commercial sex with men in the past 6 months. Sex behaviors with women were evaluated by the following questions: total number of female sexual partners, age at first anal sex with women and condom use with women.

Participants were asked if they had ever taken any drugs (including ecstasy, heroin, marijuana, opium, crystal meth, methamphetamine, cocaine, sedatives or stimulants, among others) and the frequency of taking drugs in the past month. In addition, participants were asked about their HIV testing history and whether they knew the test results.

To assess the willingness of PrEP, respondents were provided a brief description of PrEP (i.e., PrEP is a daily medication that people who do not have HIV take to prevent becoming infected with HIV; PrEP is taken before someone is exposed to HIV) and then asked to select a response on a 5-point Likert scale (1 = definitely not, 2 = probably not, 3 = not sure, 4 = probably will, 5 = definitely will). The responses were then dichotomized, whereby choices 1, 2, and 3 were classified into the “unwilling to use PrEP” group, and choices 4 and 5 were classified into the “willing to use PrEP” group.

### Sample size

The sample size was calculated using Power Analysis and Sample Size software (version 11.0.7; PASS, NCSS, LLC). Based on existing reports, the proportions of willingness to use PrEP range from 19.1% to 67.8% [22, 23], and the average of the lower and upper proportions is 43.45%. The two-sided 95% CI and margin of error were 4%, and the estimated sample size was 613. Considering a nonresponse rate of 20%, the final sample size was 613/(1–0.2) = 767. A total of 854 participants were contacted, of whom 829 were eligible and completed the survey.

### Statistical analysis

All statistical analyses were performed using SPSS version 23.0 (SPSS Inc., Chicago, IL, USA). Descriptive statistics, including the frequency, percentage, mean and standard deviation (SD), were reported. Chi-square tests and t-tests were used to compare differences in demographics, HIV/AIDS knowledge, sexual behaviors, drug use and HIV testing history between MSM who were willing and those who were unwilling to use PrEP. Variables with a p-value < 0.05 in univariate analysis were selected and further explored in a multivariate logistic regression model to evaluate the factors associated with willingness to use PrEP. Odds ratios (ORs) and 95% confidence intervals (95% CIs) were calculated. A two-tailed p < 0.05 was considered statistically significant.

## Results

### Participant characteristics

A total of 854 participants were enrolled in this study. All participants were tested for HIV screening; 25 were positive and excluded from the study. Thus, 829 eligible MSM were included in the final analysis. Among eligible MSM, 251 (30.28%, 95% CI 27.3–33.4) reported that they were willing to use PrEP for HIV prevention.

The majority of the participants were younger than 35 years old (81.2%); the mean age was 28.5 (± 9.0). More than half of the participants were registered local residents (57.3%), and 43.7% were of Han ethnic origin. Over one-fifth of respondents were freelancers, and 69.6% had a college or higher level of education. The majority of participants reported their sexual orientation as homosexual (76.2%), and 72.7% of them had never been married. Those who reported their sexual orientation as bisexual were more likely to be willing to use PrEP than those who reported being homosexual (*χ*^*2*^ = 9.498, *P* = 0.002). In addition, 5.8% self-reported a history of STDs other than HIV. The sample sources were mainly peer introduction (37.9%) and network recruitment (30.0%) (Table 1).

**Table 1:** Background characteristics and willingness to use PrEP

Variables	Total (N = 829) n (%)	Unwilling to use PrEP (n = 578) n (%)	Willing to use PrEP (n = 251) n (%)	χ^*2/t*^	p-value
Age group(years)				1.452	0.484
15–24	342 (41.2)	242 (41.9)	100 (39.8)		
25–34	323 (39.0)	228 (39.4)	95 (37.9)		
≥ 35	164 (19.8)	108 (18.7)	56 (22.3)		
Household registration				2.920	0.087
Local (Liuzhou)	475 (57.3)	320 (55.4)	155 (61.8)		
Nonlocal	354 (42.7)	258 (44.6)	96 (38.2)		
Ethnicity				46.581	< 0.001
Zhuang	315 (38.0)	238 (41.2)	77 (30.7)		
Han	362 (43.7)	210 (36.3)	152 (60.5)		
Others	152 (18.3)	130 (22.5)	22 (8.8)		
Occupation				3.974	0.410
Students	159 (19.2)	109 (18.9)	50 (19.9)		
Company Employees/government staff	155 (18.7)	117 (20.2)	38 (15.2)		
Factory workers	167 (20.1)	115 (19.9)	52 (20.7)		
Freelancers	201 (24.3)	141 (24.4)	60 (23.9)		
Others	147 (17.7)	96 (16.6)	51 (20.3)		
Education level				6.323	0.042
Middle school or below	123 (14.8)	96 (16.6)	27 (10.8)		
High school	129 (15.6)	94 (16.3)	35 (13.9)		
College or above	577 (69.6)	388 (67.1)	189 (75.3)		
Marital status				3.430	0.180
Never married	603 (72.7)	428 (74.0)	175 (69.7)		
Married	175 (21.1)	120 (20.8)	55 (21.9)		
Divorced/widowed	51 (6.2)	30 (5.2)	21 (8.4)		
Personal monthly income(RMB)					
< 1500	137 (16.5)	110 (19.0)	27 (10.8)	9.193	0.027
1500–3000	195 (23.5)	134 (23.2)	61 (24.3)		
3001–4500	347 (41.9)	236 (40.8)	111 (44.2)		
> 4500	150 (18.1)	98 (17.0)	52 (20.7)		
Sexual orientation				9.498	0.002
Homosexual	632 (76.2)	458 (79.2)	174 (69.3)		
Bisexual	197 (23.8)	120 (20.8)	77 (30.7)		
History of STDs other than HIV				5.840	0.016
Yes	48 (5.8)	26 (4.5)	22 (8.8)		
No	781 (94.2)	552 (95.5)	229 (91.2)		
Recruitment source				121.027	< 0.001
Peer introduction	314 (37.9)	220 (38.1)	94 (37.4)		
Network recruitment	249 (30.0)	202 (34.9)	47 (18.7)		
Gay venues	111 (13.4)	30 (5.2)	81 (32.3)		
Others	155 (18.7)	126 (21.8)	29 (11.6)		
HIV/AIDS knowledge scores				0.369	0.544
< 4	616 (74.3)	433 (74.9)	183 (72.9)		
≥ 4	213 (25.7)	145 (25.1)	68 (27.1)		

### HIV/AIDS knowledge

The mean score of the HIV/AIDS knowledge scale was 3.0 (± 0.92), ranging from 0 to 6. Only 25.7% of the 829 MSM participants scored 4 or higher, and 74.3% scored below 4 points. There was no significant difference in willingness to use PrEP by HIV/AIDS knowledge (*χ*^*2*^ = 0.369, *P* = 0.544) (Table 1).

### Sexual behavior characteristics

In terms of homosexual behaviors, 90.1% of the participants reported having two or more male sex partners in their lifetime, with 11.8% having 20 or more male sex partners. More than 90% of the participants reported ever having anal intercourse with male sex partners, and the mean age of first anal sex with a man was 22.64 years old (± 6.34). Approximately half (46.8%) of the participants had two or more male anal sex partners in the past month, and 27.5% did not use a condom during their last anal sexual encounter. A total of 89.0% of participants reported ever having oral sex with a man, with 44.6% having two or more male oral sex partners in the past month. Only a few participants (5.1%) stated having commercial sex with a man in the last 6 months, whereas 40.4% reported that they had casual sex other than commercial sex in the past 6 months. Regarding sexual behaviors with women, approximately 45.0% reported having one or more female sex partners in their lifetime, and the mean age of first sex with a woman was 22.15 years old (± 3.90). More than half of the participants (56.6%) reported that they had not used condoms during their last sexual encounter with a woman (Table 2).

**Table 2:** Sexual behavior characteristics and willingness to use PrEP

Variables	Total (N = 829) n (%)	Unwilling to use PrEP (n = 578) n (%)	Willing to use PrEP (n = 251) n (%)	χ^*2/t*^	p-value
Number of male sex partners in lifetime				5.340	0.254
1	82 (9.9)	64 (11.1)	18 (7.2)		
2–5	404 (48.7)	287 (49.7)	117 (46.6)		
6–10	157 (19.0)	102 (17.6)	55 (21.9)		
11–20	88 (10.6)	60 (10.4)	28 (11.2)		
> 20	98 (11.8)	65 (11.2)	33 (13.1)		
Ever had anal sex with a man				1.743	0.187
Yes	774 (93.4)	544 (94.1)	230 (91.6)		
No	55 (6.6)	34 (5.9)	21 (8.4)		
Age of first anal sex with a man (years)	22.64 ± 6.34	22.57 ± 6.62	22.80 ± 5.61	0.474	0.636
Number of male anal sex partners in the past month				1.136	0.768
≤1	412 (53.2)	287 (52.7)	125 (54.4)		
2–5	309 (39.9)	222 (40.8)	87 (37.8)		
6–10	33 (4.3)	21 (3.9)	12 (5.2)		
> 10	20 (2.6)	14 (2.6)	6 (2.6)		
Condom use at last anal sex with a man				2.679	0.102
Yes	561 (72.5)	385 (70.8)	176 (76.5)		
No	213 (27.5)	159 (29.2)	54 (23.5)		
Ever had oral sex with a man				9.213	0.002
Yes	738 (89.0)	502 (86.9)	236 (94.0)		
No	91 (11.0)	76 (13.1)	15 (6.0)		
Number of male oral sex partners in the past month				5.669	0.129
≤ 1	355 (55.4)	246 (56.4)	109 (53.2)		
2–5	245 (38.2)	167 (38.3)	78 (38.0)		
6–10	28 (4.4)	18 (4.1)	10 (4.9)		
> 10	13 (2.0)	5 (1.2)	8 (3.9)		
Had commercial sex in the past 6 months				0.350	0.554
Yes	42 (5.1)	31 (5.4)	11 (4.4)		
No	787 (94.9)	547 (94.6)	240 (95.6)		
Had casual sex other than commercial sex in the past 6 months				19.179	< 0.001
Yes	335 (40.4)	262 (45.3)	73 (29.1)		
No	494 (59.6)	316 (54.7)	178 (70.9)		
Number of female sex partners in lifetime				3.849	0.427
0	456 (55.0)	323 (55.9)	133 (53.0)		
1	227 (27.4)	163 (28.2)	64 (25.5)		
2–5	127 (15.3)	80 (13.8)	47 (18.7)		
6–10	11 (1.3)	7 (1.2)	4 (1.6)		
> 10	8 (1.0)	5 (0.9)	3 (1.2)		
Age of first sex with a woman (years)	22.15 ± 3.90	22.16 ± 3.92	22.12 ± 3.86	0.085	0.933
Condom use at last sex with a woman				0.472	0.492
Yes	145 (43.4)	93 (42.1)	52 (46.0)		
No	189 (56.6)	128 (57.9)	61 (54.0)		

### Drug use and HIV test history

Overall, 12.2% (101/829) of the participants reported that they had ever used drugs. Participants reported using drugs once in the past month (median 1, IQR: 0–2 times). Approximately one-third (32.1%) had never been tested for HIV. Among the 563 participants who had a history of HIV testing, 95.2% (536/563) reported that they knew the test results. Those who had a history of HIV testing were more likely to be willing to use PrEP than those who did not have such a history (*χ*^*2*^ = 21.356,* P* < 0.001).

### Factors associated with willingness to use PrEP

In univariate analysis, ethnicity, education level, monthly income, sexual orientation, history of STDs, recruitment source, ever having oral sex with a man, having casual sex other than commercial sex in the past 6 months and history of HIV testing were significantly associated with willingness to use PrEP.

All variables associated with willingness to use PrEP in univariate analyses were included in a multivariate logistic regression model, the results of which showed that compared to other ethnic groups, Zhuang (aOR = 3.172, 95% CI 1.696, 5.934) and Han (aOR = 8.785, 95% CI 4.722, 16.343) ethnic-origin individuals were more likely to be willing to use PrEP. Compared to participants recruited in other ways, those recruited through peer introduction (aOR = 3.584, 95% CI 2.104, 6.106) and gay venues (aOR = 22.940, 95% CI 11.608, 45.053) were more likely to be willing to use PrEP. In addition, a higher education level (college or above: aOR = 1.881, 95% CI 1.082, 3.268; reference group: middle school or below), ever having oral sex with a man (aOR = 1.949, 95% CI 1.013, 3.749) and a history of HIV testing (aOR = 2.341, 95% CI 1.552, 3.532) were significantly and positively associated with a willingness to use PrEP. Nonetheless, having casual sex in the past 6 months (aOR = 0.381, 95% CI 0.261, 0.557) was significantly and negatively associated with a willingness to use PrEP (Table 3).

**Table 3:** Logistic regression analysis of factors associated with willingness to use PrEP

Variables	Univariate analysis		Multivariate analysis	
	Crude OR(95% CI)	p-value	Adjusted OR(95% CI)	p-value
Ethnicity				
Others	1.000		1.000	
Zhuang	1.912 (1.137–3.215)	0.015	3.172 (1.696–5.934)	< 0.001
Han	4.277 (2.600–7.036)	< 0.001	8.785 (4.722–16.343)	< 0.001
Education level				
Middle school or below	1.000		1.000	
High school	1.324 (0.743–2.357)	0.341	1.252 (0.633–2.475)	0.518
College or above	1.732 (1.092–2.746)	0.020	1.881 (1.082–3.268)	0.025
Personal monthly income (yuan)				
< 1500	1.000			
1500–3000	1.855 (1.104–3.115)	0.020		
3001–4500	1.916 (1.189–3.089)	0.008		
> 4500	2.162 (1.261–3.705)	0.005		
Sexual orientation				
Homosexual	1.000			
Bisexual	1.689 (1.208–2.362)	0.002		
History of STDs other than HIV				
No	1.000			
Yes	2.040 (1.133–3.673)	0.018		
Recruitment source				
Others	1.000		1.000	
Peer introduction	1.856 (1.160–2.971)	0.010	3.584 (2.104–6.106)	< 0.001
Network recruitment	1.011 (0.605–1.689)	0.967	1.197 (0.693–2.068)	0.518
Gay venues	11.731 (6.557–20.989)	< 0.001	22.940(11.608–45.053)	< 0.001
Ever had oral sex with a man				
No	1.000		1.000	
Yes	2.382 (1.340–4.233)	0.003	1.949 (1.013–3.749)	0.046
Had casual sex other than commercial sex in the past 6 months				
No	1.000		1.000	
Yes	0.495 (0.360–0.680)	< 0.001	0.381 (0.261–0.557)	< 0.001
History of HIV test				
No	1.000		1.000	
Yes	2.250 (1.587–3.189)	< 0.001	2.341 (1.552–3.532)	< 0.001

## Discussion

In this study, demographic characteristics, including Han and Zhuang ethnic origins and pursuing college or higher education, were related to a willingness to use PrEP; sample source from peer introduction and gay venues was also related to a willingness to use PrEP, as were a history of HIV testing and having oral sex with a man. However, having causal sex in the past 6 months was significantly and negatively associated with a willingness to use PrEP.

The level of willingness to use PrEP reported herein is higher than that in a study conducted in Shanghai [22] and lower than that in Chengdu’s study [24]. Possible reasons include the period of the current study being after the Shanghai study and before Chengdu’s study and that the promotion and pilot study of PrEP in China has been gradually expanded in recent years. Another possible explanation is due to different sampling techniques. In our study, participants were included from several sampling sources, potentially resulting in a proportion difference compared with the other studies. Compared to studies based on PrEP-implemented settings, the willingness to use PrEP was lower among MSM in Liuzhou, China. Indeed, studies from the US have reported a willingness to use PrEP ranging from 46.1% to 61.0% [25, 26]. Our finding was also lower than that in studies conducted in Scotland and Spain [27, 28]. Unsurprisingly, Liuzhou is an economically undeveloped city located in southwest China, where the stigmatization of MSM has led to a lack of sufficient HIV and PrEP knowledge. In general, it is necessary to develop strategies to increase PrEP knowledge and reduce HIV stigma to motivate MSM to engage in novel HIV prevention methods [29].

Our analysis suggests that those of Zhuang and Han ethnic origins are more likely to be willing to use PrEP than other ethnic groups. A potential reason is that Liuzhou is a mountainous city, and most ethnic minorities live in rural areas and have a low level of education. Furthermore, due to the influence of traditional culture, most of them are reluctant to disclose their MSM identity. This study also found that MSM who graduate from college or have a higher education level had a greater willingness to use PrEP. This finding suggests that higher education has a significant impact on willingness to use PrEP. A previous study revealed that participants with higher education were more likely to have better knowledge of STDs and their prevention [30]. Similarly, MSM who have a higher education might have better access to health-related information. In Brazil, MSM reported that learning about PrEP online positively influenced their willingness to use it [31]. This finding highlights the need to increase access to PrEP-related knowledge among less-educated individuals.

In this study, we found that participants recruited through peer introduction were more willing to use PrEP. Overall, peers were more likely than health care workers to influence the behaviors of fellow group members. It has been reported that peer education is an effective method that increases HIV-related knowledge [32], and a meta-analysis revealed that peer-led interventions increased HIV testing among MSM [33]. Linking in peer groups might encourage MSM to discuss HIV prevention strategies with their friends. In Berlin, Germany, 90% of MSM reported already being aware of PrEP; among them, 61.7% of MSM obtained the knowledge from their friends or acquaintances [34]. Similarly, a recent study revealed that PrEP willingness among MSM is related to previous PrEP awareness [28]. Our findings support recommendations for using peer groups as a potentially effective medium for the dissemination of PrEP messaging throughout China.

Gay venues are mostly used for sourcing male sex partners by MSM, and it is a place where high-risk behaviors occur. In China, HIV incidence is growing quickly among MSM, especially those who frequently visit gay venues [35]. We found that participants recruited from gay venues were more likely to be willing to use PrEP. In London, men who frequented gay venues were more likely to report an impact on STD knowledge than those who did not [36]. The association of gay venues and PrEP willingness might be related to a higher risk perception among those who visit more frequently, as well as to a higher exposure to prevention messages from gay communities at meeting venues.

Furthermore, ever having oral sex with a man was associated with a willingness to use PrEP. The association between having oral sex and PrEP willingness might be related to a higher risk perception among MSM. Indeed, a previous study reported that higher perceived HIV risk was associated with a greater willingness to use PrEP [37]. In Nigeria, MSM who engaged in oral sex reported a high prevalence of oropharyngeal STDs [38]. Similarly, a study conducted by Templeton et al. [39] found that MSM who often engaged in insertive oro-anal sex were more likely to have oropharyngeal gonorrhea than MSM who never engaged in this behavior. According to Cornelisse et al. [40], the majority of MSM with urethral gonorrhea acquired their infection from the rectum or pharynx of their partner. Moreover, having STDs increases the risk of HIV acquisition.

The results of this study showed that having casual sex with men in the past 6 months was associated with lower willingness to use PrEP. Although further studies are needed to understand the association, it can be suggested that PrEP might be perceived as less important among MSM who engage in casual sex. Having sex with a casual male partner might increase HIV acquisition risk because it is an unplanned sexual encounter, and it might be challenging to know a partner’s serostatus. HIV serostatus disclosure might serve as a way to reduce HIV acquisition and transmission risk and might help MSM make informed decisions about safe sexual practices [41]. In a previous study, condom-less anal sexual intercourse increased among those with casual partners in the past 3 months [42]. Therefore, this population is at high risk of HIV acquisition, and it is necessary to identify whether the PrEP-related knowledge, awareness and stigma described in similar studies [23, 43] may be related to this perception.

In contrast, MSM who had undergone HIV testing in the past showed a willingness to use PrEP, which was similar to a study conducted in Hong Kong [44]. In Liuzhou, different social media technologies and on-site campaigns have been used to encourage MSM to visit CDCs, medical institutions and local CBOs to obtain free HIV testing as well as professional counseling from staff during the testing process. HIV-positive individuals screened by hospitals and CBOs are referred to CDCs for HIV confirmation and CD4 testing, and antiretroviral therapy is initiated in due course. Since 68% of our participants had been tested for HIV in the past, it is possible that these MSM might have been counselled on the efficacy of PrEP to decrease HIV transmission among them. HIV counseling and testing are the first step in making MSM aware of their serostatus. It is also important to facilitate early access to treatment, enabling the success of biomedical interventions, including prevention [45]. A study by Werner et al. [34] revealed that 14% of MSM received knowledge about PrEP from counseling centers. Similarly, in the US, the overall use of PrEP was high when offered in sexually transmitted diseases clinics and a community health center [46]. These facilities might be an ideal setting to promote PrEP. In summary, this study provides important implications for HIV prevention among MSM regarding the implementation of PrEP by combining it with other behavioral approaches. However, this study had several limitations. (1) Participants were recruited only from the Liuzhou area; therefore, the results might not be generalizable to all MSM in China. (2) Sexual behaviors were assessed by participant self-reporting and might be subject to recall bias. (3) The reasons for unwillingness to use PrEP were not assessed. Despite these limitations, this is the first quantitative analysis of PrEP willingness and associated factors among MSM in Liuzhou, China. Additionally, this is the first study conducted in HIV-negative MSM in the study area. Furthermore, the participants of our study were recruited from several sampling sources, providing a diverse sample of various experiences.

## Conclusions

The findings of this indicate that willingness to use PrEP among MSM remains low and that many of them engage in high-risk behavior. Hence, there is an urgent need to increase awareness of PrEP among MSM and to increase their knowledge about HIV prevention. In addition, Chinese public health authorities should prioritize the implementation of PrEP, which may reduce new HIV infection cases among MSM and HIV-related medical costs.

## Data Availability

The datasets used and/or analyzed during the current study are available from the corresponding author on reasonable request.

## Abbreviations

PrEP: Pre-exposure prophylaxis
HIV: Human immunodeficiency virus
AIDS: Acquired immune deficiency syndrome
MSM: Men who have sex with men
US: United States
STIs: Sexually transmitted infections
ART: Antiretroviral therapy
WHO: World Health Organization
CDC: Center for Disease Control and Prevention
CBOs: Community-based organizations
STDs: Sexually transmitted diseases
SD: Standard deviation
ORs: Odd ratios
CI: Confidence intervals
IQR: Interquartile range
aOR: Adjusted odds ratio
